# EHMTI-0233. Clinical characteristics of migraine patients with dopaminergic gene polymorphisms RS1611115

**DOI:** 10.1186/1129-2377-15-S1-B35

**Published:** 2014-09-18

**Authors:** K Skorobogatykh, J Azimova, A Sergeev, E Klimov, G Tabeeva, N Fokina, L Korobeynikova, Z Kokaeva

**Affiliations:** 1 University Headache Clinic, University Headache Clinic, Moscow, Russia; 2 Department of Genetics, Lomonosov Moscow State University, Moscow, Russia; 3 Department of Genetics, Vavilov Institute of General Genetics of Russian Academy of Sciences, Moscow, Russia

## Introduction

The dopaminergic system plays a major role in migraine. Dopamine beta hydroxylase (DBH) is responsible for maintaining dopamine-to-norepinephrine ratio implicated in migraine pathophysiology. We aimed to look for association of polymorphisms in dopaminergic genes in genetic susceptibility to migraine in Russian population. In the present study DBH polymorphisms rs1611115 was selected.

The aim of this study was to determine whether the polymorphisms rs1611115 in DBH gene influenced any particular symptoms of the disease.

## Methods

We have analyzed clinical characteristics of 128 patients with migraine (22 patients with migraine with aura and 106 patients with migraine without aura), according to the ICHD-II (2003)) taking into account their genotypes of TT variant of DBH. Genotyping was done using polymerase chain reaction (PCR).

## Results

We have shown that the T-allele carriers (46,9%) as compared to the CC genotype patients (53,1%), have more severe coarse of migraine. A significant association was shown (number of acute medication per month (p=0.04), more severe grade of medication overuse (p=0.001), presence of allodinia (p=0.03) and prodrpomal period (p=0.001).

## Conclusions

Thus, according to our data, the T-allele in rs1611115 is significantly associated with certain clinical characteristics of migraine.

No conflict of interest.

